# Response to: “Utility of endogenous 4β-hydroxycholesterol as a biomarker to assess cytochrome P 450 3A (CYP3A) activity: not quite ready for prime time”

**DOI:** 10.1007/s00228-022-03387-y

**Published:** 2022-09-13

**Authors:** Kine Eide Kvitne, Kristine Hole, Espen Molden, Ida Robertsen

**Affiliations:** 1 Section for Pharmacology, Pharmaceutical Biosciences, Department of Pharmacy, University of Oslo, Blindern, P.O. Box 1068, 0316 Oslo, Norway; 2 Center for Psychopharmacology, Diakonhjemmet Hospital, Oslo, Norway; 3 Department of Life Sciences and Health, Oslo Metropolitan University, Oslo, Norway

We thank Tung and Ma for their interest in our recently published paper presenting correlations between 4β-hydroxycholesterol (4βOHC) concentrations and three other CYP3A metrics, including intestinal and hepatic CYP3A4 protein concentrations and microsomal activity, and in vivo midazolam pharmacokinetics in primarily a population with obesity, to assess 4βOHC’s potential as an endogenous CYP3A4 biomarker [1]. In the letter to the editor, our understanding is that Tung and Ma argue that correlation coefficients are unsuitable for the validation of probe drugs or biomarkers such as 4βOHC. They are also concerned that the results presented may lead to inappropriate use of 4βOHC in other studies, particularly studies evaluating CYP3A-mediated drug-drug interactions.

First, we agree that correlation coefficients have certain limitations and that predictive performance via assessment of bias and precision should be included in the validation of phenotyping biomarkers and/or probe drugs. However, the nature of our study was exploratory, and as such, correlations analyses were performed, which are useful to assess associations between variables. One of the major strengths of our study was the ability to investigate the relationship between 4βOHC concentrations and both CYP3A4 protein expression and microsomal activity in paired liver and jejunum samples. This provides novel information, which is lacking in the literature and is actually suggested to be one of several validation criteria for phenotyping metrics [2].

Indeed, we do not believe that the results presented in our study may lead to inappropriate use of 4βOHC as a biomarker, but rather provide increased knowledge on when or how 4βOHC may be utilized as a CYP3A4 biomarker. Tung and Ma claim that there have been concerns regarding the utility of 4βOHC as a CYP3A4 biomarker, e.g., due to the uncertainty with regard to the intestinal contribution in 4βOHC formation and systemic level [3]. We have previously reported that intake of grapefruit juice, an inhibitor of intestinal CYP3A4, had no impact on 4βOHC to cholesterol ratio [4], hence showing that variability in intestinal CYP3A4 activity does not reflect 4βOHC levels. An important point that Tung and Ma seem to have missed is that the results of our current study support that 4βOHC reflects hepatic CYP3A4 activity, but not intestinal. Thus, we have clarified that 4βOHC is only appropriate as hepatic CYP3A4 biomarker.

Tung and Ma comment on the lack of correlation between 4βOHC and systemic midazolam clearance as an argument against the value of 4βOHC as CYP3A4 biomarker. While midazolam is considered to be the gold standard CYP3A probe drug, it is reported to have a highly variable extraction ratio in the literature [5], and hence midazolam clearance may not reflect hepatic CYP3A activity in all patient populations, such as patients with obesity having an increased hepatic blood flow. Thus, we believe the discrepancy between 4βOHC and systemic midazolam clearance in our study was attributed to the fact that midazolam behaved as a medium to high extraction ratio drug, and thus clearance is also dependent on hepatic blood flow. This hypothesis is supported by the fact that there was no correlation between systemic midazolam clearance and hepatic microsomal CYP3A4 activity. Accordingly, midazolam does not seem to be optimal as a CYP3A4 biomarker in patients with obesity. This is supported by previous studies showing that midazolam clearance appears to be influenced by hepatic blood flow in patients with obesity (6, 7). Thus, 4βOHC may be superior to midazolam as a hepatic CYP3A4 metric in patients with obesity, but not necessarily in other patient groups.

To date, the perfect CYP3A4 biomarker does not exist and any information obtained by the use of such metrics should be interpreted with care. However, our study suggests that 4βOHC may serve as a valuable measure to obtain information on hepatic CYP3A4 activity in clinical studies.

## Data Availability

Not applicable as no data were used.
